# Genome-Wide Analysis of Wheat GATA Transcription Factor Genes Reveals Their Molecular Evolutionary Characteristics and Involvement in Salt and Drought Tolerance

**DOI:** 10.3390/ijms24010027

**Published:** 2022-12-20

**Authors:** Xuan Du, Yuxia Lu, Haocheng Sun, Wenjing Duan, Yingkao Hu, Yueming Yan

**Affiliations:** Beijing Key Laboratory of Plant Gene Resources and Biotechnology for Carbon Reduction and Environmental Improvement, College of Life Science, Capital Normal University, Beijing 100048, China

**Keywords:** wheat, GATA transcription factor, evolutionary characteristics, expression pattern, protein–protein docking, salt and drought tolerance

## Abstract

GATA transcription factor genes participate in plant growth, development, morphogenesis, and stress response. In this study, we carried out a comprehensive genome-wide analysis of wheat GATA transcription factor genes to reveal their molecular evolutionary characteristics and involvement in salt and drought tolerance. In total, 79 *TaGATA* genes containing a conserved GATA domain were identified in the wheat genome, which were classified into four subfamilies. Collinear analysis indicated that fragment duplication plays an important role in the amplification of the wheat GATA gene family. Functional disproportionation analysis between subfamilies found that both type I and type II functional divergence simultaneously occurs in wheat GATA genes, which might result in functional differentiation of the *TaGATA* gene family. Transcriptional expression analysis showed that *TaGATA* genes generally have a high expression level in leaves and in response to drought and salt stresses. Overexpression of *TaGATA62* and *TaGATA73* genes significantly enhanced the drought and salt tolerance of yeast and Arabidopsis. Protein–protein docking indicated that TaGATAs can enhance drought and salt tolerance by interacting between the DNA-binding motif of GATA transcription factors and photomorphogenesis-related protein TaCOP9-5A. Our results provided a base for further understanding the molecular evolution and functional characterization of the plant GATA gene family in response to abiotic stresses.

## 1. Introduction

Wheat (*Triticum aestivum* L.) is one of the widely cultivated grain crops around the world and has better adaptability to adverse conditions, such as salinity, drought, and low temperature [1]. As two important abiotic stressors, salinity and drought have serious effects on crop yield and grain production. Plants generate a large amount of reactive oxygen species (ROS), and cells continue to be in a state of dehydration under such stress conditions. This causes damage to photosynthetic organs and tissues and serious inhibition of plant growth and metabolism. In addition, salt stress also causes accumulation of Na^+^ and Cl^–^ in plant cells and produces ion toxicity. 

Plants have a complex detoxification network that involves the participation of transcription factors (TFs). When plants suffer from external stress, the transduction of hormones, Ca^2+^, and other signaling pathways is activated. When stress signals reach the nucleus, transcription factors conduct transcription remodeling [2]. The late embryogenesis abundant (LEA) protein superfamily can provide osmotic protection to cells and plays important roles in the accumulation of organic osmolytes. Tonoplast-localized Na^+^/H^+^ exchanger 1 (NHX1) and plasma-membrane-localized SALT OVERLY SENSITIVE 1 (SOS1) can detoxify high intracellular Na^+^ concentrations in time [3]. Transcription factors serve as important players in maintaining normal cellular metabolism by regulating these critical downstream mediators.

It is known that transcription factors can activate or inhibit gene expression to further control plant growth and the environmental response through transcriptional regulation [4]. Some transcription factors, such as NAC, MYB, WRKY, and GATA, can participate in the regulation of responses to various abiotic stresses [5]. The GATA family is a huge group of transcription factors that can recognize the GATA motif and specifically bind to the promoter sequence (A/T)GATA(A/G) [6]. Most of them contain one or two highly conserved type IV zinc finger protein domains with the consensus sequence CX_2_CX_17–20_CX_2_C [7,8]. In plants, the *GATA* gene *NTL1* was firstly isolated from tobacco [9]. According to multiple sequence alignments and gene structure characterization, the GATA family can be divided into four subfamilies: A, B, C, and D. Among them, B subfamily members are further divided into two subgroups according to the specificity of their conserved domains: one has a leucine–leucine–methionine (LLM) domain at the C-terminus, and the other has a HAN (HANABATARANU) domain at the N-terminus. Studies have shown that both types of transcription factors have different biochemical activities [8].

To date, *GATA* genes have been discovered in several plant species, including 30 in *Arabidopsis* [10], 28 in rice [8], 28 in *Brachypodium distachyon* [11], and 96 in *Brassica napus* [12]. Functional studies have demonstrated that GATA transcription factors participate in seed germination [13], chloroplast development [14], plant-flowering time control [15], and signal transduction. In the auxin signal pathway, the transcription of *GATA*, *NITRATE- INDUCIBLE*, *CARBON-METABOLISM INVOLVED (GNC)*, and *GNC-LIKE (GNL)* genes was inhibited by regulating AUXIN RESPONSE FACTOR (ARF) and AUXIN/INDOLE-3-ACETIC ACID INDUCIBLE (AUX/IAA) proteins [16]. In rice, the expression of *OsGATA8* and *OsGATA23* was induced by abscisic acid (ABA) [5,17]. The GATA gene family also participates in the process of plant stress response. For example, GATA-binding motifs occur in the regulatory regions of many nitrogen assimilation genes. *GNC* and *GNL* can modify the expression of chloroplast-localized GLUTAMATE SYNTHASE (GLU1/Fd-GOGAT), which is the primary factor controlling nitrogen assimilation in plants’ green tissue [18]. GATA transcription factors are also involved in drought and salt stress responses. In *Populus*, *PdGNC1* mediates the production of NO and H_2_O_2_ to reduce stomatal closure by binding to the promoter of *PdHKX1* under drought stress [19]. In sweet potato, *IbGATA24* interacting with COP9-5A protein positively regulates drought and salt tolerance. This process is closely associated with ABA and jasmonic acid (JA) signaling pathways and the ROS-scavenging system [20]. Overexpression of *SlGATA17* enhances drought resistance of tomato by promoting the activity of the phenylpropanoid biosynthesis pathway [21]. As mentioned before, GATAs are involved in many physiological and biochemical processes as powerful and important transcription factors.

In wheat, the atypical GATA-like transcription factor *TaZIM-A1* serves as a negative regulator of flowering time [22]. A wheat LLM-domain-containing transcription factor *TaGATA1* positively modulates the immune response to *Rhizoctonia cerealis* through activating the expression of defense genes [23]. The wheat GATA gene *GAT1* can effectively regulate the metabolism of higher alcohols in *Saccharomyces cerevisiae* [24]. A recent report found that the wheat GATA gene family is involved in seed dormancy and germination [25]. Although preliminary structural characterization and expression profiling of the wheat GATA family were conducted, in-depth studies on the evolutionary characteristics and functional properties of *TaGATA* transcription factor genes involved in abiotic stress are still lacking.

In this study, we used the newly released wheat genome database (IWGSC RefSeq v2.1) to perform a comprehensive genome-wide analysis of wheat GATA transcription factor genes. We aimed to reveal the molecular evolutionary characteristics, expression profiling, and potential functions involved in salt and drought stresses by combining genomics, transcriptomics, AlphaFold protein structural prediction, and protein–protein docking.

## 2. Results and Discussion

### 2.1. Genome-Wide Identification and Phylogenetic and Structural Analysis of Wheat TaGATA Genes

**Figure 1:**
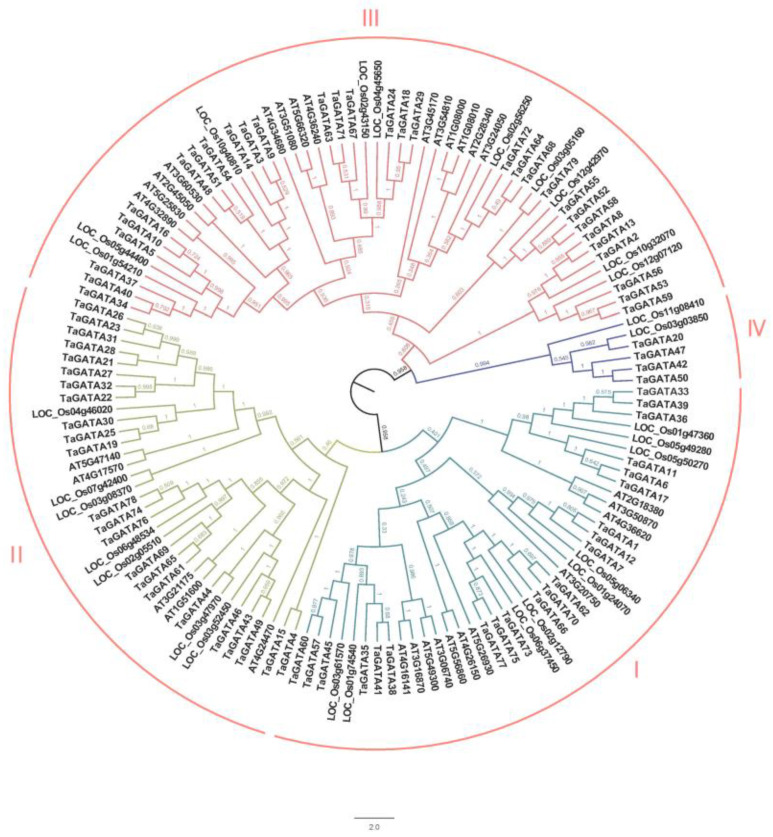
Phylogenetics of the GATA gene family among *Triticum aestivum*, *Arabidopsis thaliana*, and *Oryza sativa*. The phylogenetic tree was constructed with MEGA 6 using the NJ-method.

According to the latest wheat genome database (IWGSC RefSeq v2.1), a total of 79 wheat GATA transcription factor genes were identified via BlastP, which were further confirmed by an HMMER search (Table S1). GATA proteins had 197–642 amino acids with a molecular weight from 21.66 to 66.29 kDa and an isoelectric point from 4.98 to 9.78. Multiple sequence alignment of 137 GATA protein sequences from three plant species was used to construct a phylogenetic tree, including 79 sequences in wheat, 30 in Arabidopsis, and 28 in rice (Figure 1). Based on the topological structure and a previous report [8], the 137 GATA protein sequences were divided into four subfamilies (clades I–IV), and all of them contained a conserved GATA domain. Among them, clade III had the largest number of GATA genes and contained 55 members distributed in all three plant species. Clade I with 41 members and clade II with 35 members were also distributed in three plant species. Clade IV contained six GATA members only distributed in monocotyledons: two in rice and four in wheat.

**Figure 2:**
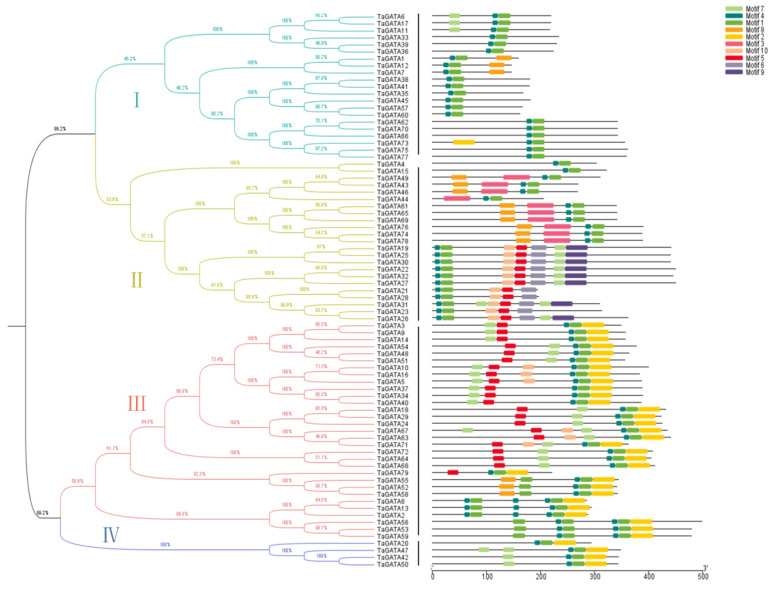
The motif structure analysis of TaGATA proteins. Four subfamilies of TaGATAs classified with MEGA6 software. The protein length (number of amino acid residues) displayed at the bottom.

Four clades of TaGATAs (Figure 2 Left) and their motif distribution features (Figure 2 Right) were analyzed with the MEME program. A total of 10 motifs were identified, and they showed a distinct distribution in different subfamilies. Most of the clade I members contained the conserved motif 1 and motif 4, while motifs 2, 7, and 8 were also present. The members of clade II included all motifs except motif 2. Some motifs, such as motifs 3, 6, and 9, were specifically present in this subfamily. The remaining motifs 5, 7, 8, and 10 showed changes in different subfamilies, suggesting that these motifs might cause functional differentiation of wheat GATA genes. All members in clades III and IV had conserved motifs 1, 2, and 4. Except for TaGATA73 from clade I, motif 2 only occurred frequently and specifically in clades III and IV. In addition, motif 7 was also abundant in both clades III and IV. All 79 TaGATA proteins contained a conserved GATA zinc finger domain CX_2_CX_17-20_CX_2_C, which might be vital in light responsiveness [26]. Both motif 1 and motif 4 were detected in the 79 TaGATAs, demonstrating their high conservation during the evolutionary process of the wheat GATA gene family.

### 2.2. Chromosomal Distribution and Collinearity Analysis of TaGATA Genes

**Figure 3:**
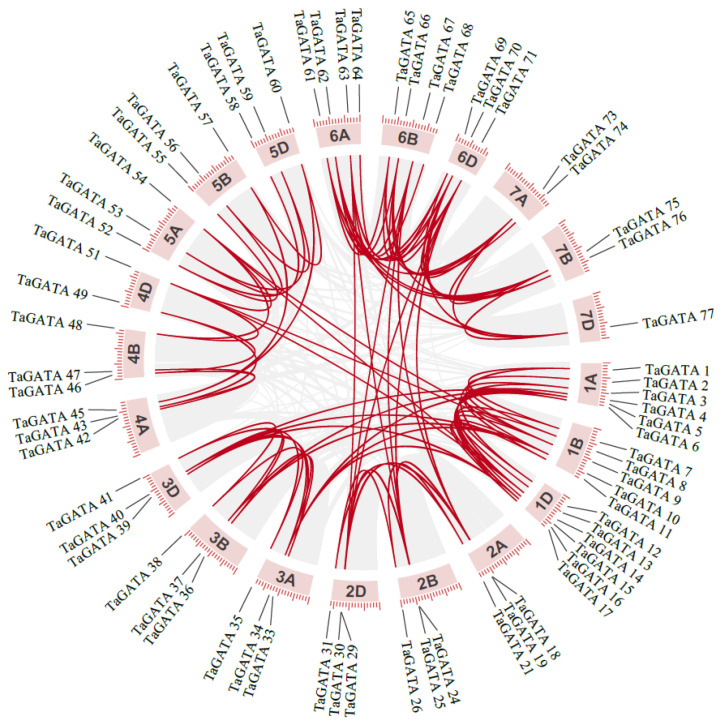
The chromosome distribution and collinearity of wheat *GATA* genes.

The chromosomal distribution of wheat GATA genes was analyzed according to IWGCS RefSeq v2.1. As shown in Figure S1, 79 *TaGATA* genes were unequally located on 21 chromosomes, with a relatively even distribution on three subgenomes: 29 on chromosome A and 25 on chromosomes B and D, respectively. Most of the 21 chromosomes contained 2-6 TaGATA genes (Figure 3).

During the evolution process, duplicated genes may be completely lost or retained, and the retained copies of genes may subsequently experience different evolutionary fates [27]. In this study, we found that gene duplication events also occur in the wheat GATA gene family. Collinearity analysis showed that 84 pairs of *TaGATA* genes have a collinearity relationship (Figure 3). Most of the *TaGATAs* had orthologous genes on A/B/D chromosomes, such as *TaGATA21* (Chr 2A), *TaGATA26* (Chr 2B), and *TaGATA31* (Chr 2D). The wheat GATA gene family was mainly amplified by tandem replication and fragment replication, such as *TaGATA18* (Chr 2A), *TaGATA24* (Chr 2B), *TaGATA29* (Chr 2D), and *TaGATA71* (Chr 6D). These results are generally consistent with previous reports [28,29].

### 2.3. Functional Disproportionation and Positive Selection Analysis of TaGATA Genes

The mutation of amino acid sites is closely related to the functional disproportionation of repetitive genes, which occurs frequently and accumulates in large quantities [30,31]. Functional disproportionation analysis of three *TaGATA* subfamilies (I, II, and III; the results of subfamily IV were erroneous and were excluded) showed that type I functional disproportionation occurs between clade I and clade II and between clade I and clade III (Table 1). Eight sites were identified between clade I and clade II (37K, 40G, 66A, 67E, 72A, 78P, 81A, and 86N), while three sites were identified between clade I and clade III (39C, 72A, and 86N). For type II functional disproportionation, eight, six, and three sites were found between clade I and clade II, clade I and clade III, and clade II and clade III, respectively. However, the θ_2_ value was less than 0, suggesting that type II functional disproportionation is not significant among these subfamilies.

Posterior probability test results showed that five amino acid sites (37K, 66A, 67E, 72A, and 86N) were simultaneously identified in both type I and type II functional disproportionation, indicating that these amino acid sites are more likely to change in terms of the evolution rate and amino acid physicochemical properties. 

During the evolution process, new genes produced by gene duplication may be lost and new functions may evolve and be retained under positive selection pressure. The CODEML program in PAML v4.4 software was used to calculate the selection effects that occurred during the evolution of the wheat GATA protein family, and the M0 and M3 and the M7 and M8 models were used for analysis. The results showed that no key positive selection loci were identified in the TaGATA family (Table 2), indicating that TaGATAs experience a strong purifying selection pressure with high conservation during the evolutionary process.

### 2.4. Three-Dimensional Structure Characterization, Coevolution Analysis, and Subcellular Localization of TaGATA Proteins

**Figure 4:**
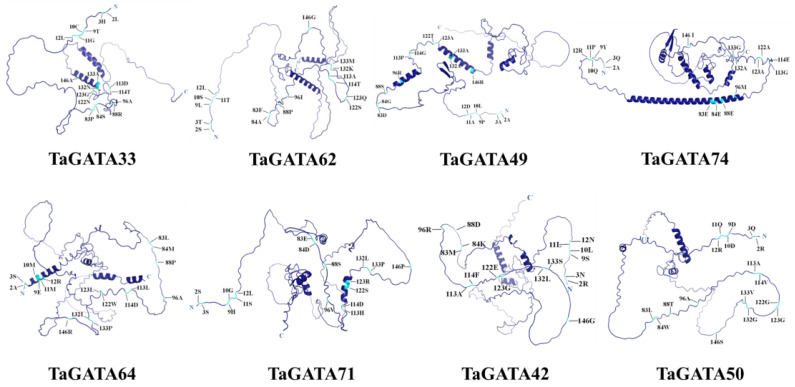
Three-dimensional structures of eight TaGATA proteins from different subfamilies predicted with AlphaFold2 and the distribution of coevolution sites.

Eight representative TaGATA proteins (two from each subfamily) were selected to simulate their 3D structures using AlphaFold2 that can handle the missing physical context and produce accurate models in challenging cases [32]. The predicted model of TaGATA proteins was mainly composed of α-helixes, β-turns, and random coils with 234–405 amino acids (Figure 4). In general, α-helixes were widely distributed and each chosen protein had two to five α-helixes. Among them, TaGATA33 and TaGATA62 from subfamily I contained only two α-helixes. In comparison, β-turns appeared less frequently in TaGATAs, and only TaGATA49 and TaGATA74 from subfamily II had β-turn structures.

The functional disproportionation and coevolution sites were marked on the 3D structure of TaGATA proteins (Figure 4 and Figure S2). Coevolutionary sites refer to amino acid sites that play a complementary role in the evolution of protein families. If there is a structural or functional connection between different amino acid positions, changes in certain amino acid residues may lead to the substitution of amino acid residues that interact with the site in order to maintain the stability of the protein structure and function [33]. The identification of coevolutionary sites is beneficial for the annotation of protein functions as well as the mechanism research of protein interaction and adaptive variation. In this study, CAPS software was used to identify the coevolutionary sites of the wheat GATA family. Nine groups of coevolutionary sites were identified, and the residues in groups 1, 3, 4, 7, 8, and 9 were adjacent in the primary structure (Table 3). Most of them were distributed outside the DNA-binding area (Figure 4). Significant mutations in any of these amino acids may affect the coevolved protein domains. Five sites (83P, 84S, 88R, 132N, and 146A) together constituted the most complex set of coevolutionary sites, which may help to maintain the spatial structure of TaGATA proteins. These results provide new insight into the subsequent study of GATA family functional loci.

The reference sequences were TaGATA33 and TaGATA62 from clade I, TaGATA49 and TaGATA74 from clade II, TaGATA64 and TaGATA71 from clade III, and TaGATA42 and TaGATA50 from clade IV.

**Figure 5:**
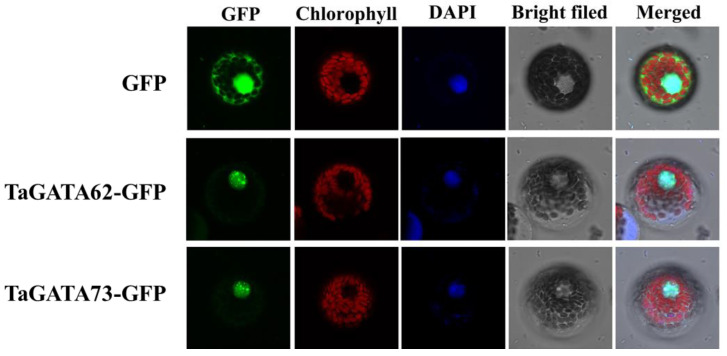
Subcellular localization of TaGATA62 and TaGATA73. Confocal images were captured with Leica SP8 fluorescence confocal microscopy (bar = 10 μm).

The subcellular localization prediction of TaGATA proteins showed that all 79 TaGATAs are located in the nucleus. To further determine the reliability of the prediction results, we selected two wheat GATA proteins, TaGATA62 and TaGATA73, and constructed 16318-TaGATA62-GFP and 16318-TaGATA73-GFP recombinant vectors to transform wheat protoplasts. As shown in Figure 5, the GFP empty vector was expressed in the cytoplasm and the nucleus, while GFP fusion proteins of TaGATA62 and TaGATA73 were only expressed in the nucleus, indicating that both transcription factors TaGATA62 and TaGATA73 are localized in the nucleus. These results were consistent with the online predictions.

### 2.5. Cis-Acting Element Analysis of TaGATA Genes

In this study, we selected 2000 bp upstream promoter sequences of wheat GATA genes for *cis*-acting element analysis. In total, 1012 *cis*-acting elements related to hormone and stress response were detected in 79 *TaGATA* genes through the PlantCARE website (Table 4). These elements were classified into 10 categories: TCA-element, TGA-element, TGACG-motif, CGTCA-motif, ABRE, LTR, GC-motif, ARE, MBS, and TC-rich repeat. The distribution of these elements had no apparent subfamily specificity. Among them, ABRE, CGTCA-motif, and TGACG-motif were the most abundant elements among hormone-responsive-related elements, being 239, 203, and 199 in number, respectively. They accounted for 63% of all elements, and their main function was involved in ABA and MeJA responsiveness. The stress-related elements involved in environmental stress responses, such as low temperature, drought, and hypoxia stresses, were also abundant. In particular, ARE elements involved in anoxic inducibility accounted for 34%. The existence of these *cis*-acting elements could play an important role in hormone regulation and environmental stress resistance.

### 2.6. RNA-seq Expression Profiling of TaGATA Genes

**Figure 6:**
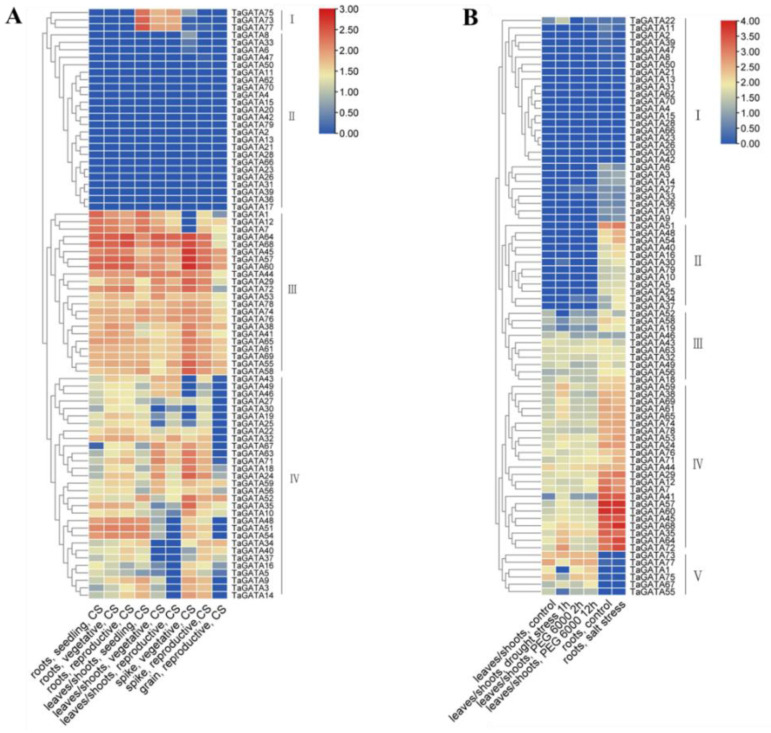
Transcriptional expression analysis of *TaGATA* genes by RNA-seq. (**A**) Expression profiling of *TaGATA* genes in different organs. (**B**) Expression profiling of *TaGATA* genes under abiotic stresses.

The expression profiling of 79 *TaGATA* genes in different wheat organs and in response to abiotic stresses was analyzed using the wheat RNA-Seq database (Figure 6). Wheat *GATA* genes were divided into four expression patterns (I–IV) according to the tissue expression preference and expression level. In general, *TaGATA* genes in patterns I and II showed a lower expression level in almost all organs except for *TaGATA73*, *TaGATA75*, and *TaGATA77* with high expression in leaves and shoots in different growing stages. Genes in pattern III and most of the genes in pattern IV were highly expressed in different organs (Figure 6A). *TaGATA* genes from different subfamilies showed expression specificity in different organs. Most of the genes from subfamily I were highly expressed in leaves and shoots, but more than half of the genes were not expressed in roots, spikes, and grains. Most genes from subfamily II were expressed in roots, leaves, shoots, and spikes, while some showed low expression in all organs, such as *TaGATA21*, *TaGATA23*, and *TaGATA28*. The expression of subfamily III genes could be detected in multiple organs and had no obvious tissue expression preference, while the genes in subfamily IV generally showed a lower expression level in all tissues. These results indicated that homologous genes from the same subfamily generally have a similar expression pattern in different organs.

Under various abiotic stresses, *TaGATA* genes showed five expression patterns (I–V), including drought, PEG-simulated drought, and salinity (Figure 6B), which contained 28, 12, 10, 23, and 6 genes, respectively. The genes in pattern I had a low expression level under stress treatments, except for *TaGATA22* with upregulated expression under drought stress. In pattern II, the genes were upregulated after salt treatment, while those in pattern III had no obvious expression changes under abiotic stresses. The genes in pattern IV responded to drought stress but had no significant expression changes under salinity stress. Pattern V only contained six genes, in which *TaGATA1* and *TaGATA55* showed downregulation under drought treatment and the remaining genes had no significant expression differences.

### 2.7. Transcriptional Expression Patterns of TaGATA Genes by qRT-PCR

**Figure 7:**
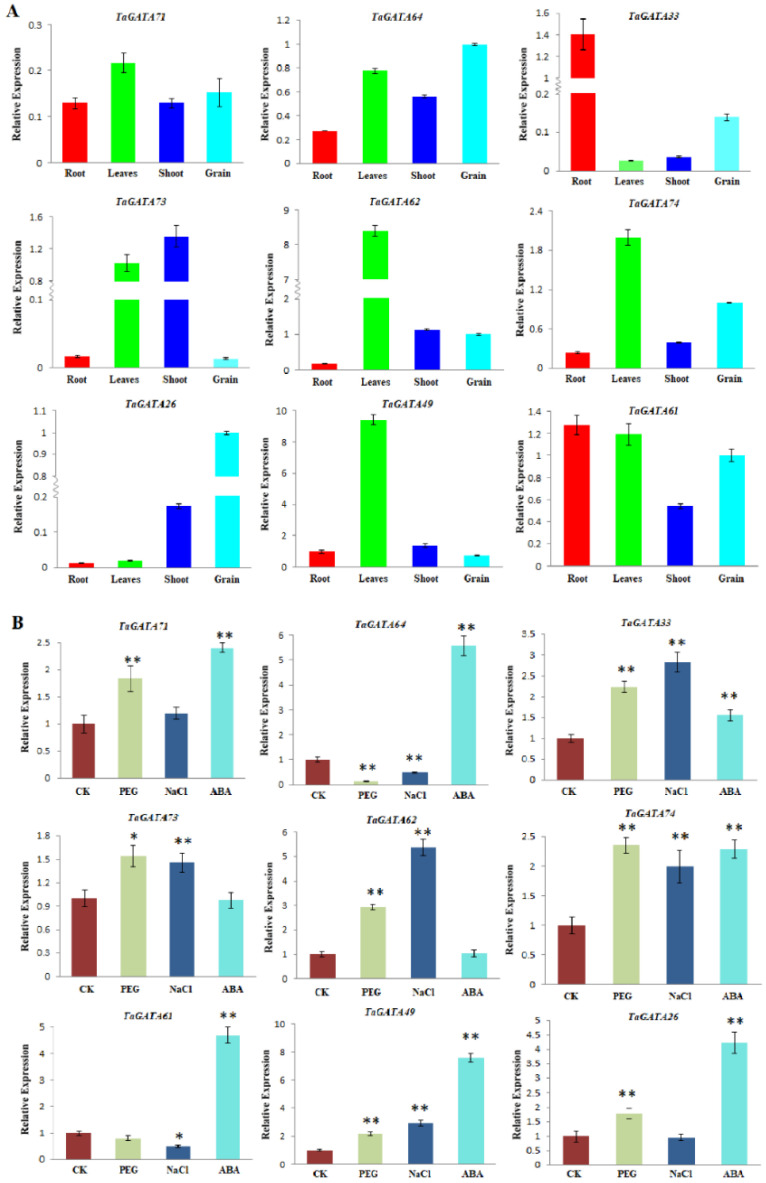
Expression analysis of nine wheat *TaGATA* genes by qRT-PCR. (**A**) Expression patterns of *TaGATA* genes in different organs. (**B**) Expression patterns of *TaGATA* genes under various abiotic stresses. Error bars represent standard deviations for three replicates. * *p* < 0.05; ** *p* < 0.01.

Nine representative *TaGATA* genes were selected from different subfamilies to further detect their expression changes in different organs and in response to abiotic stresses (Figure 7). The results showed that *TaGATA* genes are generally highly expressed in leaves, except for *TaGATA26* and *TaGATA33* with a lower expression level. *TaGATA61*, *TaGATA64*, and *TaGATA71* had a higher expression level in all four organs. Some genes were highly expressed in a particular organ but had a lower expression level in other organs, such as *TaGATA33* in roots, *TaGATA26* in grains, and *TaGATA49* and *TaGATA62* in leaves. In addition, *TaGATA73* had an expression preference in both leaves and shoots. These results were consistent with RNA-seq expression analysis, but some genes showed differences in expression specificity, such as TaGATA74 in roots and shoots, probably due to the differences in wheat materials and growing stages.

Wheat *GATA* genes generally showed significantly expression changes when subjected to drought, salt, and ABA treatments (Figure 7B). Five *TaGATA* genes (*TaGATA33*, *TaGATA49*, *TaGATA71*, *TaGATA73*, and *TaGATA74*) showed upregulated expression in response to three abiotic stresses, while two genes were upregulated under two abiotic stresses: *TaGATA26* under PEG and ABA stresses and *TaGATA62* under PEG and salt stresses. In addition, *TaGATA61* and *TaGATA64* were significantly upregulated under ABA treatment but downregulated under PEG and salt stresses.

As important regulators, transcription factors can activate or inhibit gene expression. GATAs are an important class of transcription factors and can control many biological processes, such as plant growth, development, and environmental response, through transcriptional regulation [26,28]. For example, peanut *GATA9* was downregulated under low-temperature treatment [34]. In sweet potato, IbGATA24 interacting with COP9-5A protein and positively regulated drought and salt tolerance [20]. In this study, we also found through qRT-PCR analysis that *TaGATA* genes are responsive to salt, ABA, and PEG simulation drought stress (Figure 7B). These results were closely related to *cis*-acting elements present in *TaGATA* genes. ABRE, MBS, and TC-rich repeats are the promoter elements involved in ABA, drought, and stress responsiveness, respectively. These environmental responsive elements present in *TaGATA* genes could participate in regulating *TaGATA* gene expression and enhance salt and drought resistance, such as MBS elements in *TaGATA62*, *TaGATA73*, and *TaGATA74*; TC-rich repeats elements in *TaGATA64* and *TaGATA74*; and ABRE elements in all *TaGATA* genes.

### 2.8. Overexpression of TaGATA62 and TaGATA73 in Yeast and Arabidopsis Enhanced Drought and Salt Tolerance

**Figure 8:**
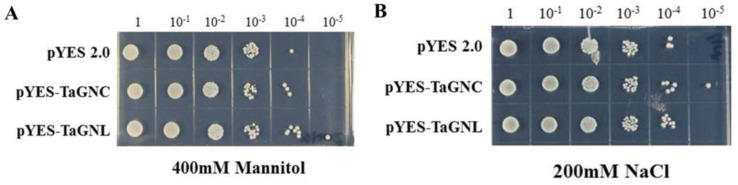
Growth states of transgenic yeast on selective media. The pYES2 empty vector was used as a negative control. (**A**) Yeast grew on SD/–Ura/400 mM mannitol medium. (**B**) Yeast grew on SD/–Ura/200 mM NaCl medium.

In this study, we used the BY4741 yeast strain to explore the heterologous expression and stress resistance of *TaGATA62* and *TaGATA73* genes. pYES2 (empty vector), pYES2-TaGATA62, and pYES2-TaGATA73 were transferred into yeast and cultured on SD/-Ura/400 mM mannitol and SD/-Ura/200 mM NaCl solid medium after serial dilution. The results showed that when the bacterial concentration was 10^−4^ on SD/-Ura/400 mM mannitol medium, the growth state of both pYES2-TaGATA62 and pYES2-TaGATA73 yeast cells was clearly better than that of pYES2 empty vector yeast cells. pYES2-TaGATA73 yeast cells at 10^−5^ bacterial concentration continued to grow (Figure 8A). pYES2-TaGATA62 and pYES2-TaGATA73 yeast cells on SD/-Ura/200 mM NaCl medium also had a better growth state compared to pYES2 yeast cells (Figure 8B). These results indicated that *TaGATA62* and *TaGATA73* can enhance drought and salt tolerance of the BY4741 yeast strain.

**Figure 9:**
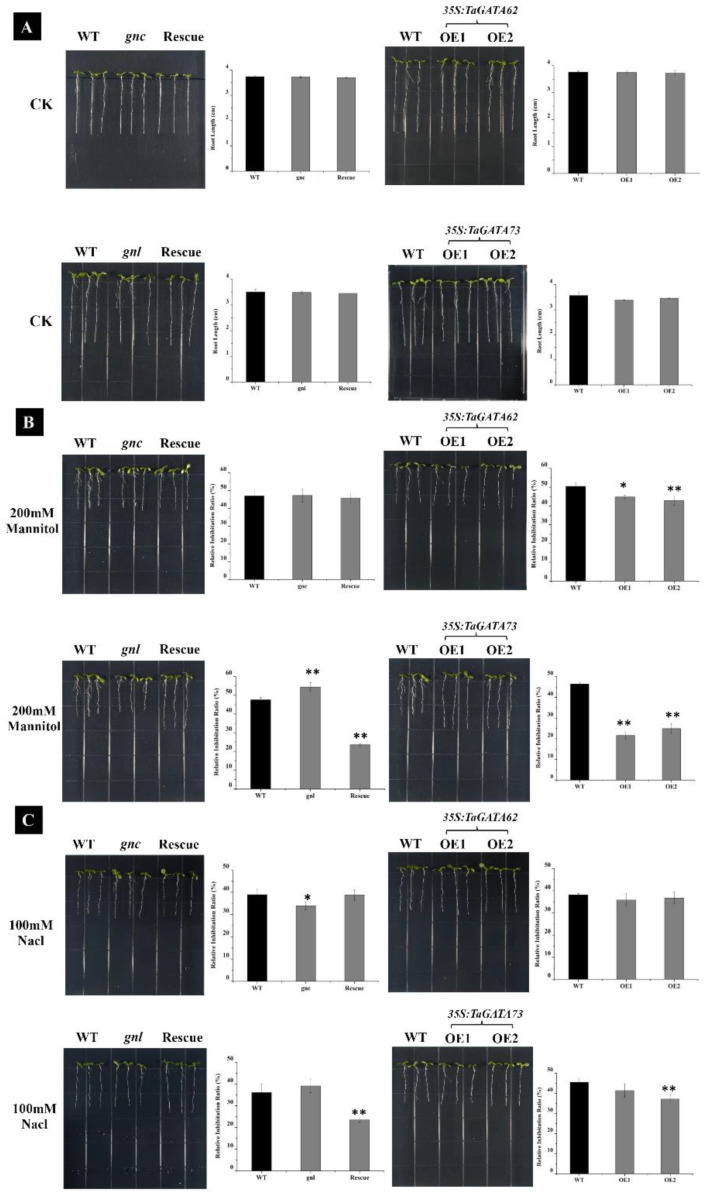
Effects of 200 mM mannitol and 100 mM NaCl treatments on the root growth of *TaGATA62* and *TaGATA73* transgenic Arabidopsis plants. (**A**) Phenotype of 35s:TaGATA62 and 35s:TaGATA73 transgenic plants under normal conditions. (**B**) Phenotype of 35s:TaGATA62 and 35s:TaGATA73 transgenic plants under 200 mM mannitol treatment. (**C**) Phenotype of 35s:TaGATA62 and 35s:TaGATA73 transgenic plants under 100 mM NaCl treatment. Mean  ±  SD of data from three independent biological replicates. *gnc*, the loss-of-function mutant of TaGATA62; *gnl*, the loss-of-function mutant of TaGATA73; OE, the overexpression lines of TaGATA62 and TaGATA73. * *p* < 0.05; ** *p* < 0.01.

The genetic transformation of *TaGATA62* and *TaGATA73* in Arabidopsis were further conducted to explore their function in response to drought and salt stresses. The loss-of-function mutants (*gnc/TaGATA62-mutant* and *gnl/TaGATA73-mutant*) and *TaGATA62* and *TaGATA73* overexpression lines were detected through PCR analysis (Figure S3). Seven *Arabidopsis* lines, including wild type, *gnc* and *gnl* mutants, Rescue-*TaGATA62* and Rescue-*TaGATA73*, and overexpressed lines OETaGATA62 and OETaGATA73, were removed to 1/2MS medium with stress treatments after roots grew to 1.5 cm. The results indicated that all lines in the control group grew normally, while they were inhibited to varying degrees in 200 mM mannitol and 100 mM NaCl medium (Figure 9). On 200 mM mannitol medium, the root length of *gnl* mutants were shorter than those of the WT after being inhibited, while no significant changes were observed in *gnc* mutants (Figure 9B). On 100 mM NaCl medium, the root length of *gnc* mutants was longer compared to wild type and no obvious phenotypic changes were found in *gnl* mutants (Figure 9C). A complementary experiment in the mutants showed a significant reduction in root length inhibition. The primary root length of the complementing line Rescue-*TaGATA73* was 1.39 and 1.18 times more than that of the WT in 200 mM mannitol and 100 mM NaCl medium, respectively. Yet, Rescue-*TaGATA62* showed no significant morphological changes. Moreover, the resistance of overexpressed lines OETaGATA62 and OETaGATA73 was better than that of the WT on 200 mM mannitol medium. The root length of OETaGATA62 and OETaGATA73 was about 1.17 and 1.44 times more than the wild type, respectively (Figure 9B). On 100 mM NaCl medium, OETaGATA62 was inhibited to a similar extent as the WT, while OETaGATA73 grew better than the WT (Figure 9C). 

It has been reported that GATA transcription factors may take part in the response to salt and drought stresses. In tomato, overexpression *SlGATA17* enhanced drought resistance through promoting the activity of the phenylpropanoid biosynthesis pathway [21]. In sweet potato, *IbGATA24* could positively regulate drought and salt tolerance by interacting with IbCOP9-5A [20]. Our results also demonstrated that *TaGATA73* and *TaGATA62* can play important roles in drought and salt stress responses.

### 2.9. Protein Docking of TaGATAs and TaCOP9-5A

**Figure 10:**
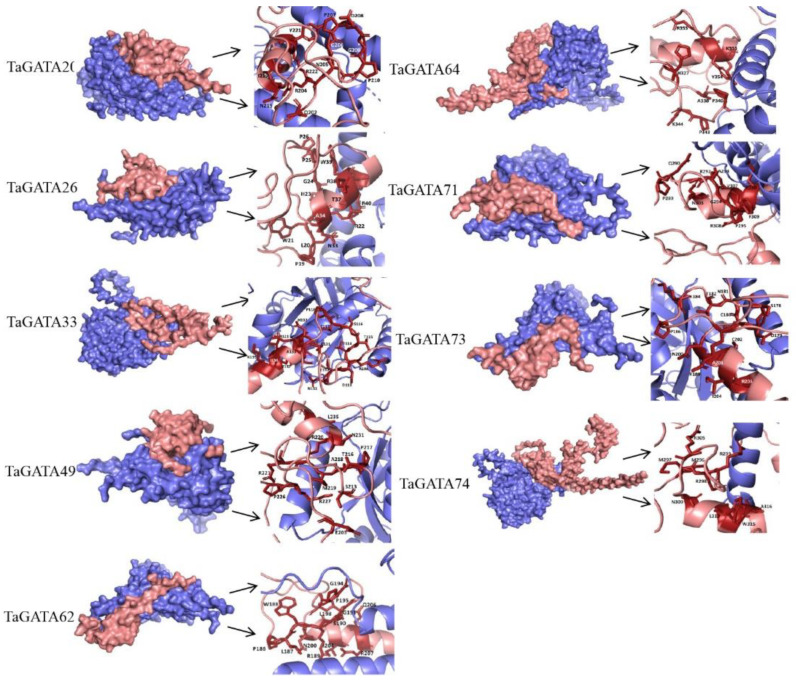
The docking modes of TaCOP9-5A with respect to nine TaGATA proteins, including TaGATA33, TaGATA62, and TaGATA73 from clade I; TaGATA26, TaGATA49, and TaGATA74 from clade II; TaGATA64 and TaGATA71 from clade III; and TaGATA20 from clade IV. The docking modes of TaCOP-5A with TaGATA proteins and the binding sites between TaCOP9-5A and TaGATA proteins that overlapped with the distribution of motif 1 and motif 4 were presented. The original three-dimensional structures were predicted with Alphafold2.

A previous study on sweet potato found that a photomorphogenesis-related protein IbCOP9-5A can interact with IbGATA24 and participate in the abiotic stress response [20]. Here, we selected nine TaGATA proteins and TaCOP9-5A, the homologous protein of IbCOP9-5A, to perform protein-docking simulation. Subcellular localization prediction with Cell-PLoc 2.0 showed that TaCOP9-5A is localized in the nucleus. The interactions between TaCOP9-5A and nine TaGATA proteins were simulated through the HDOCK website: TaGATA33, TaGATA62, and TaGATA73 from subfamily I; TaGATA26, TaGATA49, and TaGATA74 from subfamily II; TaGATA64 and TaGATA71 from subfamily III; and TaGATA20 from subfamily IV. The docking pose with the highest score was used for each interaction. The HDOCK website showed the interface residues within 5.0 Å from their interacting partner as the docking sites. The results revealed that the docking sites of different TaGATA proteins are related to the motif distribution (Table S2). In particular, the docking sites of each TaGATA protein were highly coincident with the distribution of motif 1 and motif 4. Residues marked in red in Table S2 are the sites that overlapped with the distribution of motif 1 and motif 4, which are also highlighted in Figure 10. These two motifs contained the conserved zinc finger domain CX_2_CX_17-20_CX_2_C, which is the DNA-binding region of plant GATA transcription factors [8].

The COP9 signalosome (CSN) is a conserved protein complex containing eight subunits [35] and was first discovered in *Arabidopsis* [36]. In plants, CSN is involved in the debenzylation of cullin-RING E3 ubiquitin proteases [37], phytohormone signaling [38,39], and temperature, drought, and salt stress resistance [20,40]. It is known that CSN-5A has a regulatory effect on ABA INSENSITIVE 5 (ABI5), an important transcription factor of the ABA signaling pathway [41]. In sweet potato, IbCOP9-5A interacts with IbGATA24, and they together act as a regulatory effector in the process of plant salt and drought responses [20]. In wheat, TaCSN5 also serves as a negative regulator of wheat leaf rust resistance [42].

Transcription factors play a crucial role in the regulation of plant defenses, and their interactions with regulatory proteins are an important way for the activation or repression of gene expression. For example, ANKYRIN-REPEAT PROTEIN (ANK1) in tobacco interacts with transcription factor BZI1 and co-regulates the auxin signaling pathway and pathogen stress response [43]. The transcription factor octadecanoid-responsive-Catharanthus-APETALA2-domain protein (ORC1) of tobacco can bind to MAPK and be phosphorylated to increase its own activity and then promote the expression of the downstream genes related to nicotine synthesis [44]. Histone deacetylase 19 (HDA19) binds to transcription factors AtWRKY38 and AtWRKY62 and reduces their activity, thereby inhibiting them from activating the negative regulators of plant defense [45]. These regulatory proteins modulate the activities of transcription factors and affect signal transduction pathways in plant defenses against biotic and abiotic stresses [46]. Transcription factors may also enhance their own transcriptional activity by binding to other protein factors through their DNA-binding regions. Studies have shown that the DNA-binding motif of plant transcription factor WRKY33 can interact with nuclear-encoded SIGMA FACTOR BINDING PROTEIN1 (SIB1) and SIB2, which improved its transcription efficiency [47]. In this study, the results of molecular docking indicated that TaCOP9-5A interacts with the DNA-binding motif of TaGATAs, which might enhance its DNA-binding activity to activate downstream genes and ultimately improve plant drought and salt stress tolerance. Our findings have important implications for future studies of the GATA family in wheat in terms of abiotic stress functions.

### 2.10. A Putative Transportation Regulatory Pathway of TaGATA Genes Involved in Drought and Salinity Tolerance

Previous studies have shown that ABA plays an important role in plant resistance to abiotic stresses. Plants can synthesize ABA and initiate downstream signaling processes under abiotic stress conditions, such as regulating stomatal conductance and the expression of defense genes [48,49]. ABA can also affect the expression of *GATA* genes. In rice and chickpea, *OsGATA23* and 12 *CaGATAs* were induced by ABA treatment [5,50]. Under salt and drought stress, a significantly increased ABA content and ABA-pathway-related genes *AtNCED* and *AtAAO* were detected in *IbGATA24*-overexpression transgenic lines. The leaf stomatal aperture and water loss rate in the overexpression lines were lower than those in WT lines [20]. Our qRT-PCR results also indicated that ABA can induce the expression of *TaGATA* genes (Figure 7B), which might defend against drought and salt stress through the ABA signaling pathway.

When plants suffer from hyperosmotic stress, a large number of ROS, such as H_2_O_2_, O^2−^), are generated [3]. High concentrations of ROS can easily lead to cell membrane lipid peroxidation, protein denaturation, carbohydrate oxidation, and DNA damage [51]. Thus, it is important to maintain the balance between ROS production and scavenging [52]. In tomato, overexpression of the *SlGATA17* gene can effectively alleviate the accumulation of hydrogen peroxide and O^2−^ induced by drought [21]. Under salt stress, OsGATA8 positively regulates the expression of downstream genes that play a part in the ROS-scavenging system, such as *SOD*, *CAT*, and *APX* [17].

**Figure 11:**
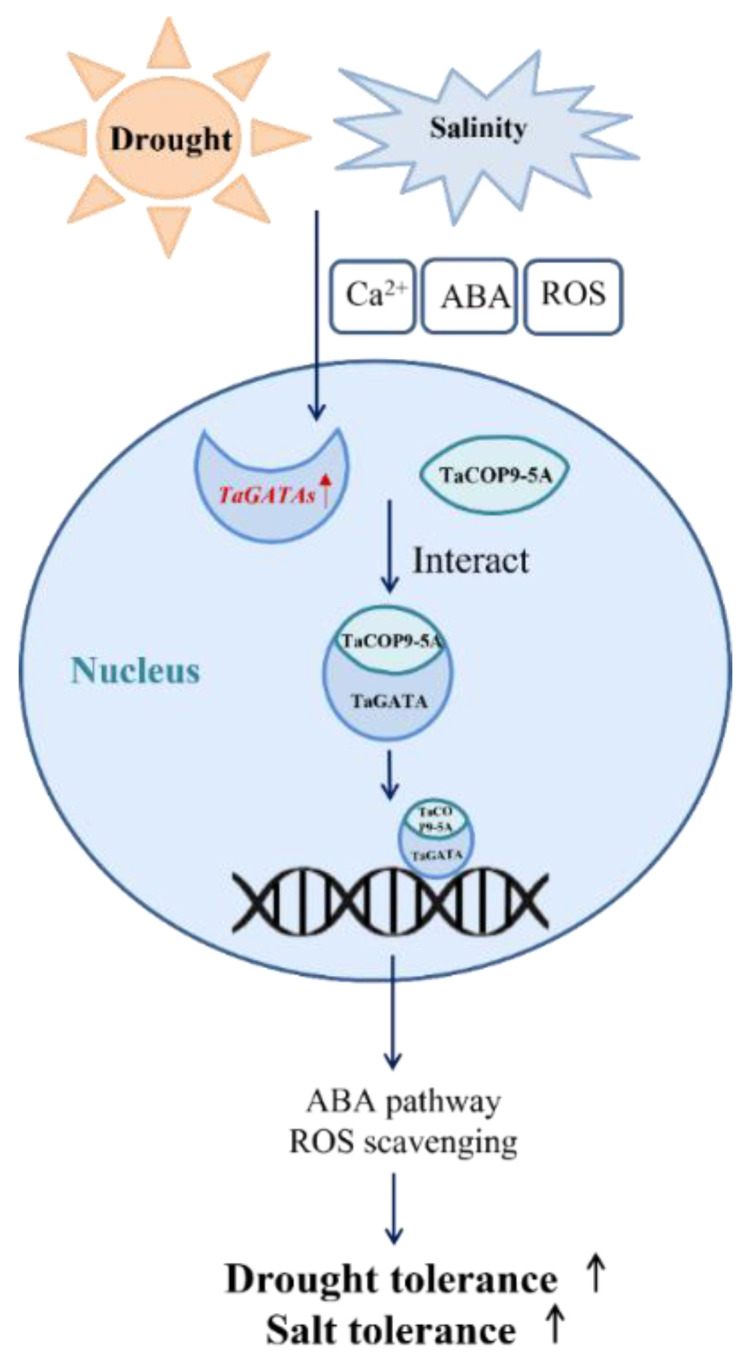
A putative transportation regulatory pathway of *TaGATA* genes involved in salt and drought tolerance.

Here, we present a putative transportation regulatory pathway to illustrate the mechanism of how *TaGATAs* defend against salt and drought stresses based on our results and previous studies (Figure 11). When plants are subjected to salt and drought stresses, Ca^2+^, ROS, and ABA signaling were activated shortly, which caused the upregulated expression of wheat *GATA* genes by stress-related *cis*-acting elements. Subsequently, GATA genes could promote the expression of downstream stress-related genes to regulate stomatal conductance and ROS scavenging and ultimately improved plant resistance. In addition, TaGATAs might interact with TaCOP9-5A to mediate the expression of ABA and ROS-scavenging-related genes, thereby enhancing salt and drought tolerance.

## 3. Materials and Methods

### 3.1. Genome-Wide Identification of TaGATA Genes 

GATA proteins from Arabidopsis were downloaded from TAIR (https://www.arabidopsis.org, accessed on 20 December 2020) and used as reference sequences for BlastP of wheat GATA proteins from the EnsemblPlants database (http://plants.ensembl.org/index.html, IWGSC RefSeq v1.0, accessed on 22 December 2020) and WheatOmics (http://wheatomics.sdau.edu.cn, IWGSC RefSeq v2.1, accessed on 4 May 2021). SMART (http://smart.embl-heidelberg.de, v9, accessed on 20 December 2020) [53] and Pfam (http://pfam.xfam.org, v33.1, accessed on 20 December 2020) [54,55] databases were used to identify the conserved domain of wheat GATA proteins. The genetic screening criteria of TaGATAs were based on the GATA domain (PF00320). The HMMER search was used to further check the *TaGATA* genes based on the conserved domain of wheat GATA proteins [56]. The HMM document of PF00320 was downloaded from the Pfam database. The coding sequences, protein sequences, and gene sequences of GATA family members were downloaded from the EnsemblPlants database for subsequent analysis.

### 3.2. Phylogenetic and Motif Analysis

GATA protein sequences in *Arabidopsis*, rice, and wheat were aligned using BioEdit, MEGA6, and Figtree software to construct the phylogenetic tree based on Tamura et al. [57]. The conserved motifs of wheat GATA proteins were identified using the MEME website (http://meme-suite.org/tools/meme, accessed on 11 January 2021) according to Bailey et al. [58]. TBtools software (v1.082) was used to visualize protein structures, and the maximum number of motifs was set to 10.

### 3.3. Collinearity Analysis and Chromosomal Distribution

Wheat genome data files were downloaded from the EnsemblPlants database, and the Super Circos and Gene Location programs in TBtools software were used to draw the collinearity graph and chromosomal distribution to analyze the duplication pattern of the wheat GATA gene family.

### 3.4. Functional Disproportionation, Positive Selection, and Coevolution Analysis

DIVERGE v3.0 software combined with posterior probability analysis was used to determine whether *GATA* genes are functionally disproportionate between different subfamilies. The CODEML program in PAML v4.4 was selected to calculate the selection effect that occurred during the evolution of the GATA family. ꞷ = 1, ꞷ < 1, and ꞷ > 1 corresponded to neutral selection, purification selection, and positive selection, respectively. Two pairs of models, M0 (one scale) and M3 (discrete) and M7 (beta) and M8 (beta and ω) were used based on previous research [59]. CAPS software was used to perform coevolution analysis and calculate the coevolution sites of the GATA protein family. PyMOL software was used to mark the selected coevolution sites on the 3D structure.

### 3.5. Three-Dimensional Structure and cis-Acting Element Analysis

The recently developed protein structure prediction website AlphaFold was used to simulate the GATA protein 3D structure [60,61,62]. PyMOL software (version 1.7.4) was used to mark the selected functional disproportionation sites on the 3D structure. The 2000 bp upstream promoter region of wheat GATA genes was obtained from the EnsemblPlants website. The obtained promoter sequences were submitted to the PlantCARE website (http://bioinformatics.psb.ugent.be/webtools/plantcare/html/, accessed on 7 June 2021) to obtain the *cis*-acting elements of *TaGATA* genes.

### 3.6. Subcellular Localization

The Cell-PLoc 2.0 website (http://www.csbio.sjtu.edu.cn/bioinf/Cell-PLoc-2, accessed on 28 July 2021) [63] was used for subcellular localization prediction. Since the 5′sequences of two target genes had no difference, the same upstream primer and different downstream primers were used to clone the target genes, and the homology arms of the subcellular localization vector were added to the primers. All the primers used are listed in Table S4. The resultant 163-TaGATA62-GFP and 163-TaGATA73-GFP plasmids were introduced into competent cell DH5α (TransGen Biotech). Plasmid transformation into wheat protoplasts was performed by using polyethylene glycol 4000 (PEG4000)-mediated transformation according to Zou et al. [64]. Protoplast cells were incubated in darkness for 24 h, and confocal images were captured with Leica SP8 fluorescence confocal microscopy.

### 3.7. RNA-seq Expression Profiling and qRT-PCR Analysis of TaGATA Genes

The expression data of *TaGATA* family members in five tissues at different periods and under abiotic stresses were obtained via the expVIP database (IWGSC RefSeq v1.1). Next, the relative expression level of each gene in the form of a heat map was obtained with tbtools software.

The seeds of wheat cultivar Zhongmai 175 were cultivated in Hoagland nutrient solution. After the seedlings grew to the three-leaf stage, samples of roots, stems, and leaves were collected and stored in liquid nitrogen for later analysis. Meanwhile, different abiotic stress treatments during seedling growth were performed, including salt stress (200 mM NaCl), simulated drought stress (20% PEG6000), and ABA stress (100 μM ABA). After 12 h of treatment, leaf samples were collected and stored in liquid nitrogen for later use.

The protocol of Cao et al. [65] was used for total RNA isolation from wheat samples and cDNA preparation. Quantitative real-time polymerase chain reaction (qRT-PCR) was conducted on the CFX96 Real-Time System with the SYBR Premix Ex Taq kit (Vazyme) using the designed specific primers (Table S4). The internal control was 18s RNA, and the reaction conditions included 40 cycles at 95 °C for 15 s, 58 °C for 15 s, and 72 °C for 10 s. The relative expression levels of wheat *GATA* genes were determined with the comparative threshold cycle (CT) method (2^−ΔΔCT^).

### 3.8. Overexpression of TaGATA Genes in Yeast and Arabidopsis

pYES2-TaGATA62 and pYES2-TaGATA73 recombinant vectors were constructed and transformed into the BY4741 yeast strain by referring to the operation manual (Clontech), then cultured to OD 2.0 in liquid glucose-Ura medium, and diluted to 6 gradients equally in multiples of 10 [66]. They were added dropwise to 200 mM NaCl and 400 mM Manitol medium, respectively, and incubated at 30 °C for 2–3 days.

1302-TaGATA62 and 1302-TaGATA73 recombinant vectors were constructed for the transformation of the GV3101 A. Tumefaciens strain, and A. Tumefaciens transformation was performed using the freeze–thaw method [67]. The transformation in *Arabidopsis* was performed using the floral dip method.

### 3.9. Identification of Arabidopsis Mutants and Transgenic Plants

*Arabidopsis* mutants *gnc* (SALK_001778C) and *gnl* (SALK_003995) were purchased from the *Arabidopsis* Biological Resource Center (ABRC) website (https://abrc.osu.edu, accessed on 9 March 2021). The seeds were sterilized and germinated in plates containing 0.6% (*w*/*v*) agar and one-fifth-strength Hoagland’s nutrient solution. After 7 days, the seedlings were moved to the soil (nutrient soil:vermiculite = 1:2) and cultivation was continued in a greenhouse. The genomic DNA of mutants was extracted according to the CTAB method and identified using the three-primer method after 14 days. LP, BP, and RP primers are shown in Table S5.

Transgenic lines include complementation lines (R-TaGATA62, R-TaGATA73) and overexpression lines (OE-TaGATA62, OE-TaGATA73). Their seeds were sterilized and germinated on 1/2MS medium containing 50 mg/L of hygromycin resistance. Genomic DNA was extracted after 3 weeks and screened by PCR.

### 3.10. Protein–Protein Docking and Binding Site Analysis

TaCOP9-5A, the wheat homologous protein of IbCOP9-5A, was identified by blasting using the EnsemblPlants website. Protein 3D structures were predicted with AlphaFold. Protein–protein docking predictions were performed using the HDOCK website (http://hdock.phys.hust.edu.cn, accessed on 25 April 2022) based on Yan et al. [68], and the docking pose with the highest score was used for each interaction result.

## 4. Conclusions

The latest wheat genome database was used to perform genome-wide identification, molecular evolution, and functional analysis of the wheat GATA transcription factor gene family. In total, 79 *TaGATA* genes were identified from the genome-wide level, and they were classified into four subfamilies (I–IV). Positive selection analysis showed that *TaGATA* genes experience strong purifying selection pressure during the evolution process. Nine groups of coevolutionary sites were identified, which might play an important role in maintaining the stability of the GATA protein structure and function. Transcription expression analysis by RNA-seq and qRT-PCR indicated that *TaGATAs* generally have high expression in leaves and in response to drought and salt stress responses. Overexpressed *TaGATA62* and *TaGATA73* in yeast and Arabidopsis enhances drought and salt tolerance. The protein–protein docking simulation showed that TaGATAs can interact with TaCOP9-5A via binding the conserved zinc finger domain CX_2_CX_17-20_CX_2_C present in motif 1 and motif 4 of TaGATAs, thereby improving the expression of the downstream genes related to abiotic stresses and enhancing salinity and drought tolerance. Our results provided new insights into the structures, evolution, and functions of the plant GATA gene family.

## Figures and Tables

**Table 1:** Functional divergence of TaGATA proteins *.

Clade 1	Clade 2	Type I			Type II	
		Ɵ_1_ ± s.e.	LRT	Sites with Qk > 0.8	Ɵ_2_ ± s.e.	Sites with Qk > 0.8
**I**	II	0.343 ± 0.099	18.075 **	**37K**, 40G, **66A**, **67E**, **72A**, 78P, 81A, **86N**	−0.097 ± 0.219	**37E**, 41D, 42D, 45Y, **66A**, **67E**, **72A**, **86N**
I	III	0.287 ± 0.110	13.339 **	39C, **72A**, **86N**	−0.045 ± 0.231	45Y, 65R, 63T, **67E**, **86N**, 87G
II	III	−0.0023 ± 0.022	0	None	−0.171 ± 0.245	63T, 65R, **66A**

* The thickened amino acid sites represent that type I and II functional divergence occurred simultaneously. All sites were mapped to the TaGATA33 reference sequence. θ_I_ and θ_II_ are functional disproportionation coefficients, which can be used to determine the degree of disproportionation occurring. LRT represents the likelihood ratio statistics, and the Qk value can detect the specific amino acid sites with functional disproportionation. ** *p* < 0.01.

**Table 2:** Positive selection analysis of the TaGATA family by using the M0 and M3 and the M7 and M8 models.

Model	np	InL	2ΔInL	Estimation Parameters	Positively Selected Sites
M0 (one-ratio)	135	−3136.437920	359.33 (M3 VS M0)	ω = 0.11010	Not allowed
M3 (discrete)	139	−2956.771985		p_0_ = 0.34018 ω_0_ = 0.00216 p_1_ = 0.30170 ω_1_ = 0.07184 p_2_ = 0.35812 ω_2_ = 0.29727	None
M7 (beta)	136	−2960.857047	0.00084 (M8 VS M7)	p = 0.36695 q = 2.71337	Not allowed
M8 (beta and ω)	138	−2960.857465		p_0_ = 0.99999 p = 0.36695 q = 2.71337 p_1_ = 0.00001 ω = 1.00000	None

**Table 3:** Coevolutionary sites of the TaGATA family *.

Groups	Coevolution Sites
1	2L, 3H
2	2L, 9T
3	9T, 10C
4	11G, 12L
5	96A, 122N
6	83P, 84S, 88R, 132N, 146A
7	113D, 114T
8	122N, 123G
9	132N, 133A

* The reference sequence was TaGATA33.

**Table 4:** Cis-acting elements analysis of TaGATAs.

	Hormone Responsive Elements					Stress-Related Elements				
**Element**	TCA-element	TGA-element	TGACG-motif	CGTCA-motif	ABRE	LTR	GC-motif	ARE	MBS	TC-rich repeats
**Amount**	34	44	199	203	239	47	79	101	54	12

## Data Availability

Not applicable.

## Supplementary Materials

The supporting information can be downloaded at https://www.mdpi.com/article/10.3390/ijms24010027/s1.

## Abbreviations

ABA	Abscisic acid
CSN	COP9 signalosome
GNC	GATA, nitrate-inducible, carbon-metabolism involved
GNL	GNC-LIKE
qRT-PCR	Real-time quantitative polymerase chain reaction
RNA-seq	RNA sequence
ROS	Reactive oxygen species
TFs	Transcription factors
