# Supplementary material for: Genome-Wide Analysis of Wheat GATA Transcription Factor Genes Reveals Their Molecular Evolutionary Characteristics and Involvement in Salt and Drought Tolerance
Source: Int J Mol Sci. 2022 Dec 20;24(1):27. doi: 10.3390/ijms24010027 (PMC9820438; doi:10.3390/ijms24010027)
Supplement: Supplementary file 1 [file ijms-24-00027-s001.zip › ijms-2034273-supplementary.docx]

**Figure S1.**  Chromosomal distribution of wheat *GATA* genes.

**
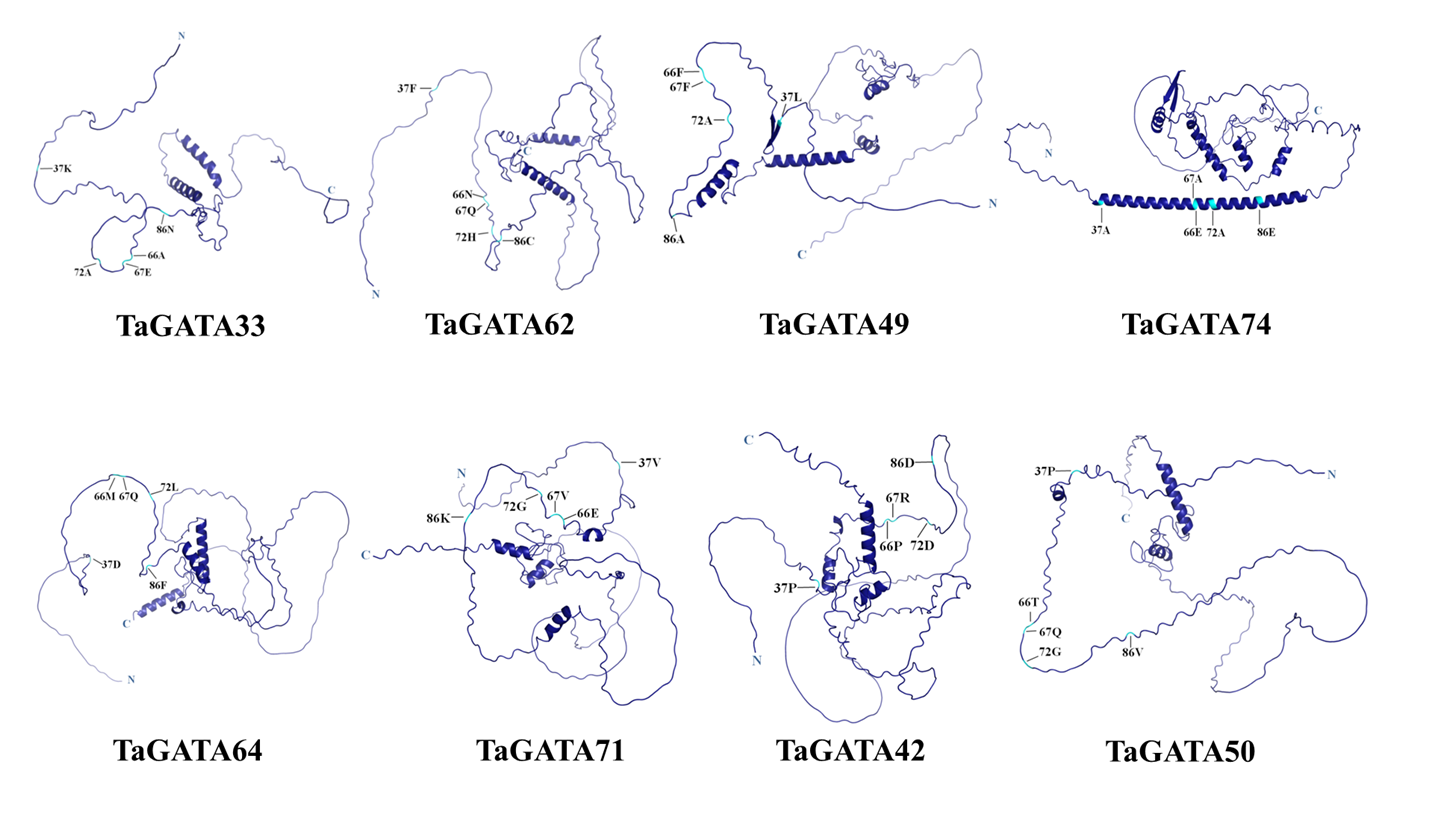
**

**Figure S2.** Three-dimensional structure with functional disproportionation sites of wheat GATA protein. The reference sequences were TaGATA33 (Clade Ⅰ), TaGATA62 (Clade Ⅰ), TaGATA49 (Clade Ⅱ), TaGATA74 (Clade Ⅱ), TaGATA64 (Clade Ⅲ), TaGATA71 (Clade Ⅲ), TaGATA42 (Clade Ⅳ) and TaGATA50 (Clade Ⅳ).

**
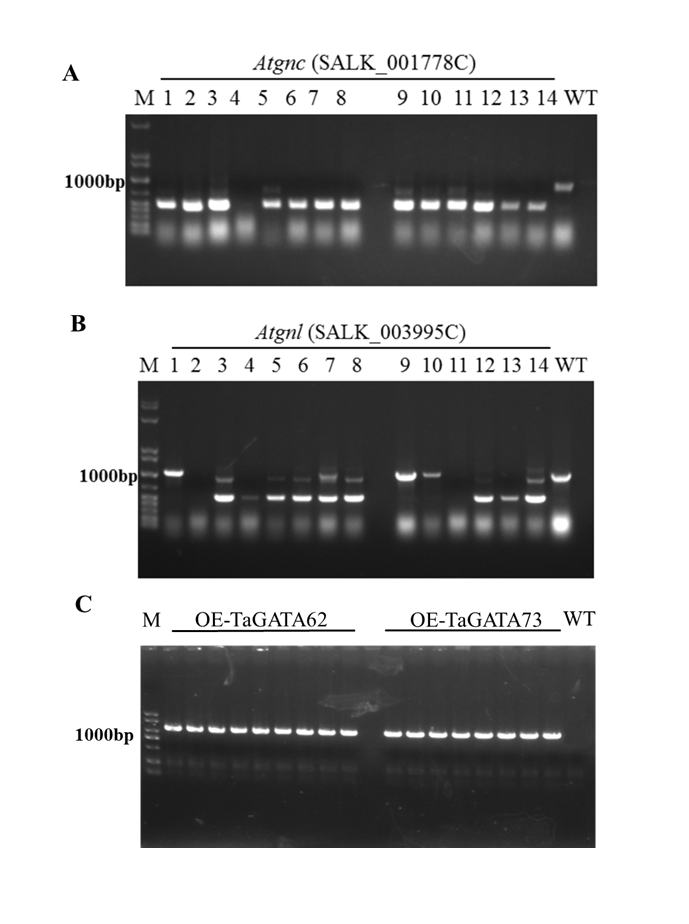
**

**Figure S3.** Identification of *Arabidopsis* mutants and overexpression lines. (A) PCR detection of *gnc* mutants. (B) PCR detection of *gnl* mutants. (C) PCR detection of OE-TaGATA62 and OE-TaGATA73.
